# Development and Application of a Short Tandem Repeat Multiplex Typing Assay for Candida tropicalis

**DOI:** 10.1128/spectrum.04618-22

**Published:** 2023-01-30

**Authors:** Bram Spruijtenburg, Merlijn H. I. van Haren, Anuradha Chowdhary, Jacques F. Meis, Theun de Groot

**Affiliations:** a Department of Medical Microbiology and Infectious Diseases, Canisius Wilhelmina Hospital, Nijmegen, The Netherlands; b Centre of Expertise in Mycology, Radboud University Medical Center/Canisius Wilhelmina Hospital, Nijmegen, The Netherlands; c Medical Mycology Unit, Department of Medical Microbiology, Vallabhbhai Patel Chest Institute, University of Delhi, Delhi, India; d Department I of Internal Medicine, University of Cologne, Excellence Center for Medical Mycology, Cologne, Germany; Institut Pasteur

**Keywords:** *Candida tropicalis*, genotyping, short tandem repeats, PCR, whole-genome sequencing

## Abstract

Candida tropicalis is a clinically important yeast that causes candidemia in humans with a high mortality rate. The yeast primarily infects immunocompromised patients, and causes outbreaks in health care facilities. Antifungal resistant isolates have been reported. We developed a short tandem repeat (STR) typing scheme for C. tropicalis to enable fast, cost-effective, and high-resolution genotyping. For the development of the typing scheme, 6 novel STR markers were selected, combined into 2 multiplex PCRs. In total, 117 C. tropicalis isolates were typed, resulting in the identification of 104 different genotypes. Subsequently, the outcome of STR typing of 10 isolates was compared to single nucleotide polymorphism (SNP) calling from whole-genome sequencing (WGS). Isolates with more than 111 SNPs were differentiated by the typing assay. Two isolates, which were identical according to SNP analysis, were separated by STR typing in 1 marker. To test specificity, the STR typing was applied to 15 related yeast species, and we found no amplification of these targets. For reproducibility testing, 2 isolates were independently typed five times, which showed identical results in each experiment. In summary, we developed a reliable and multiplex STR genotyping for C. tropicalis, which was found to correlate well to SNP calling by WGS. WGS analysis from and extensive collection of isolates is required to establish the precise resolution of this STR assay.

**IMPORTANCE** Candida tropicalis frequently causes candidemia in immunocompromised patients. C. tropicalis infections have a high mortality rate, and the yeast is able to cause outbreaks in health care facilities. Further, antifungal resistant isolates are on the rise. Genotyping is necessary to investigate potential outbreaks. Here, we developed and applied a STR genotyping scheme in order to rapidly genotype isolates with a high-resolution. WGS SNP outcomes were highly comparable with STR typing results. Altogether, we developed a rapid, high-resolution, and specific STR genotyping scheme for C. tropicalis.

## INTRODUCTION

Fungal diseases have an estimated annual death toll of 1.5 million, of which the yearly incidence of invasive candidiasis is approximately 750,000, and has been increasing for the last few decades (1–3). Invasive candidiasis has a high mortality rate, and affects primarily elderly and neonatal patients. Candida tropicalis is regarded as one of the most clinically relevant *Candida* species due to the number of infections and its virulence. C. tropicalis infections are rising and are, in some countries, the primary cause of candidiasis, surpassing the genetically related Candida albicans (4–6). The yeast is diploid with a range of virulence factors, and is considered the second most virulent *Candida* species after C. albicans (7, 8). Antifungal resistant C. tropicalis isolates are emerging against azoles and echinocandins, which makes it harder to treat infections, resulting in increased mortality (9–12).

Outbreaks caused by C. tropicalis primarily occur in intensive care units (ICUs), where patients that receive antibiotics have indwelling catheters, and undergo abdominal surgery are at high risk (13–15). Healthcare personnel surveillance suggested that cross-contamination between personnel and patients is a major risk factor (16). When an outbreak occurs, it is essential to prevent further spread of the pathogen by identifying the source and understanding the transmission route, in order to take adequate measures. If a potential outbreak is suspected, rapid and high-resolution genotyping is necessary to prevent further spread (17). Multiple genotyping methods for C. tropicalis are available, all with their own benefits and weaknesses. Currently used methods include amplified fragment length polymorphism (AFLP), multi locus sequence typing (MLST), and whole-genome sequencing (WGS) (18–24). The latter method has the best resolution, but has a long turn-around-time, which is not desired when a potential outbreak is suspected. AFLP shows often inconsistent results between different laboratories, and results are difficult to interpret. While MLST is a reproducible method, it has a lower resolution compared to other methods. Short tandem repeat (STR), or microsatellite genotyping, proved to be highly reproducible, fast, and has high-resolution (25–27). This genotyping method differentiates isolates based on highly variable repeat numbers in repeating sequences, called STRs (28). Interpretation of the results is straightforward, and can be easily implemented across different laboratories. A disadvantage of this method is that the technique has to be set up separately for each species, and a large collection of genetically variable strains is required.

Previously, 2 C. tropicalis STR genotyping assays have been developed, and applied to Chinese isolates (29, 30). These 2 typing assays consisted of 6 and 8 markers, respectively, were amplified in monoplex, and not validated with WGS SNP analyses. Here, we developed a novel C. tropicalis STR typing assay comprising of 6 novel markers, amplified in 2 multiplex PCRs, and compared its outcome with WGS SNP analysis.

## RESULTS

### Selection of STR markers and development of multiplex PCR.

Tandem repeats from the C. tropicalis IBUN-090-03567 genome were identified with Tandem Repeat Finder, and 3 trinucleotide and 3 hexanucleotide repeats were selected (Table 1). WGS data of 5 C. tropicalis isolates were mapped against the IBUN-090-03567 genome, and flanking sequences of the tandem repeat were visualized to identify conserved regions. Primers were designed on conserved regions in proximity to the STR, and screened for potential cross-dimer formation, and coupled to fluorescent probes. After amplification of the STR regions on a selected group of isolates and primer optimization, the 3 trinucleotide and 3 hexanucleotide repeats were combined into 2 PCR multiplexes, M3 and M6, respectively.

**TABLE 1:** Overview of PCR primers for selected STR loci, concentration used in multiplex PCR, details of repeat characteristics, discriminatory index, and genomic sites

PCR panel and primer name	Primer sequence (5′–3′)		Conc^*b*^ (μM)	No. of bases of primer-flanking sequence	Repeat unit	No. of repeats^*c*^		No. of alleles	*D* Value^*d*^	Intragenic/locus protein coding gene^*e*^
	Forward primer^*a*^	Reverse primer				Min	Max			
M3										
M3-a	FAM- TTGGAAGAAGTGAAATCAAAGG	ACAAGTAGATGTTCCAGCAACC	2	174	ATC	3	28	18	0.83	Carbohydrate kinase, FGGY, C-terminal
M3-b	JOE- CAAGGGCACAACAATTACAAG	GTGGTTGCATTTGAGGTGAG	1	73	CAA	7	45	25	0.90	TEA/ATTS domain superfamily
M3-c	TAMRA-AGAGGGAGACGATATTGATGTTC	ACCTATACCCGAAGCAGAGAC	5	78	TAT	5	44	22	0.90	Intragenic
M6										
M6-a	FAM- CATACTGCCTTCGGTACTGC	AATCTGCAGGTGATACCAGTTC	1	170	AGAAGC	3	46	32	0.94	Agglutinin-like protein
M6-b	JOE- GGTTCAAATACTGTCGTCCCTAC	ACCGTTTCCAGAGTCATTGC	3	195	TCTGGT	3	73	21	0.93	Hyphally regulated cell wal protein
M6-c	TAMRA- TCTTCCTCTACTTCCTCATCGAC	ATATTGAAGAATCTGATGACGTTG	5	116	CCTCTT	5	88	46	0.96	M3K1M5_CANMX

a FAM, 6-carboxyfluorescein; JOE, 4’,5′-dichloro-2’,7’-dimethoxy-fluorescein; TAMRA, 6-carboxytetramethylrhodamine.

b Conc, concentrations; forward and reverse primers are identical.

c Min, minimum; Max, maximum.

d Discriminatory power of STR assay as determined via the Simpson index of diversity.

e Reference strain is IBUN-090-03567 (GCA_019239295.1).

### STR genotyping of C. tropicalis isolates.

**FIG 1:**
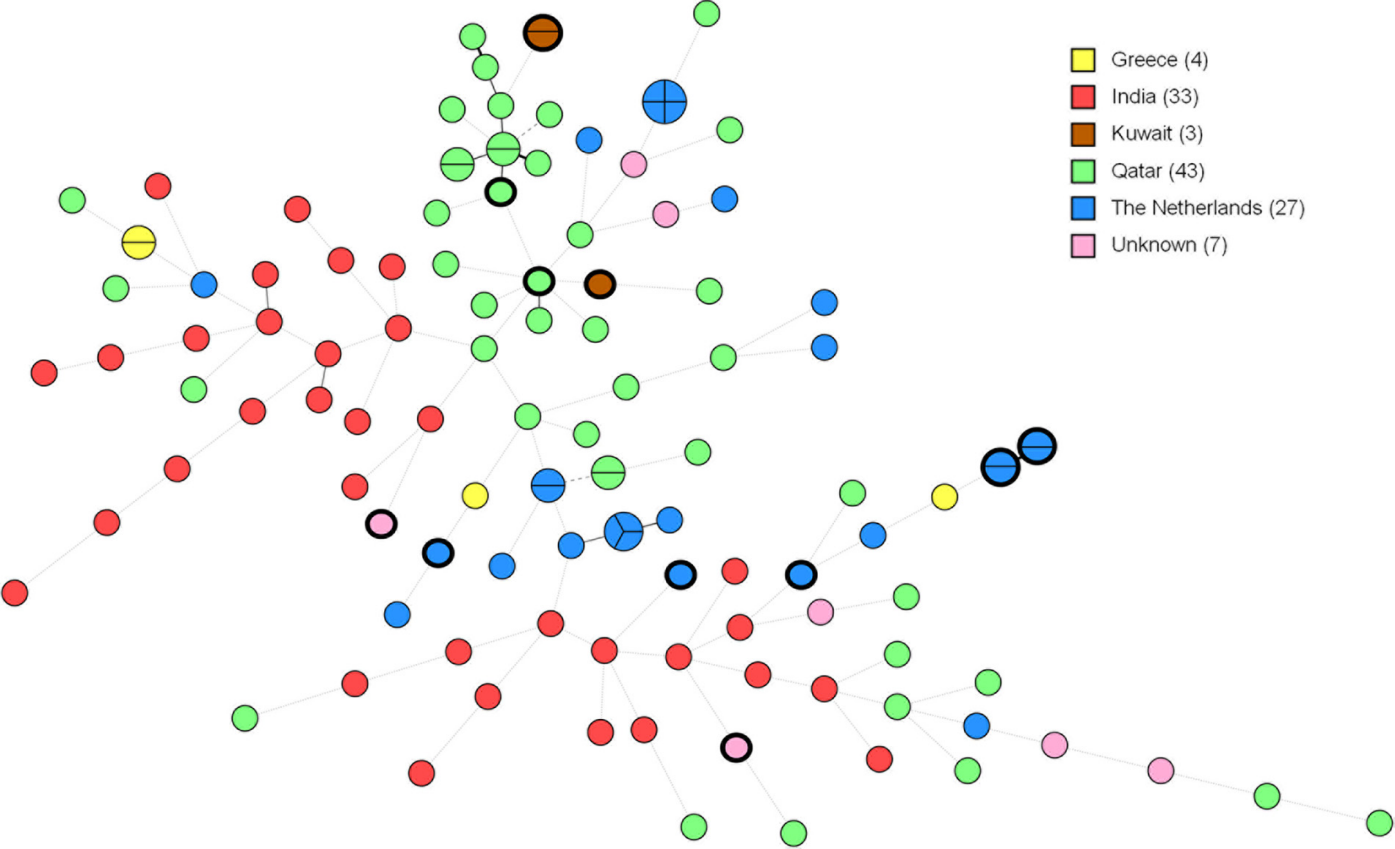
Minimum-spanning tree of 117 C. tropicalis isolates. Branch lengths indicate the similarity between isolates with thick solid lines (variation in one allele), thin solid lines (variation in two alleles), thin dashed lines (variation in three alleles), and thin dotted lines (variation in four or more alleles). Number of isolates per country are shown in the color key. Fluconazole resistant isolates are marked.

The 2 multiplex PCRs were then applied to all 117 C. tropicalis isolates, previously identified by MALDI-TOF (Table S1). An overview of the number of genotypes found, repeat characteristics, and Simpson’s index of diversity (D), which ranged from 0.83 to 0.96, is shown in Table 1. Among 117 C. tropicalis isolates, 104 different genotypes, representing 1 to 4 isolates, were identified (Fig. 1 and Fig. S1). All isolates had 1 or 2 alleles per marker, indicating these strains exhibited homozygous and heterozygous repeats. Two pairs of genotypes (35 and 36, and 97 and 98) exhibited identical STR profiles, except for a difference in zygosity in 1 marker. Two other genotypes (93 and 94) differed in 2 markers in zygosity, besides demonstrating identical copy numbers for the remaining markers. While the majority of the isolates displayed unique genotypes according to STR typing, there were some small clusters of isolates from The Netherlands, Greece, Qatar, and Kuwait. Strains in Dutch clusters originated from single patients, and were isolated on the same or different dates, which ranged from 1 day to 17 months. To test the reproducibility of the STR genotyping, DNA from isolates 10-03-02-20 and 10-08-13-91 were independently amplified five times in 5 replicate experiments. STR genotyping showed identical results in each experiment for all STR markers, demonstrating that the genotyping method is highly reproducible. The stability of the STR markers was tested by subcloning 5 colonies of the 2 previously mentioned isolates for 10 generations, after which the copy number of the markers did not change in any of the colonies, indicating high genetic stability. To test the specificity of this STR genotyping, 15 other related yeast species were analyzed by both multiplex PCRs (Table S2). None of the species tested formed PCR products after amplification, suggesting that the STR typing assay is specific for C. tropicalis.

### WGS SNP analysis.

**FIG 2:**
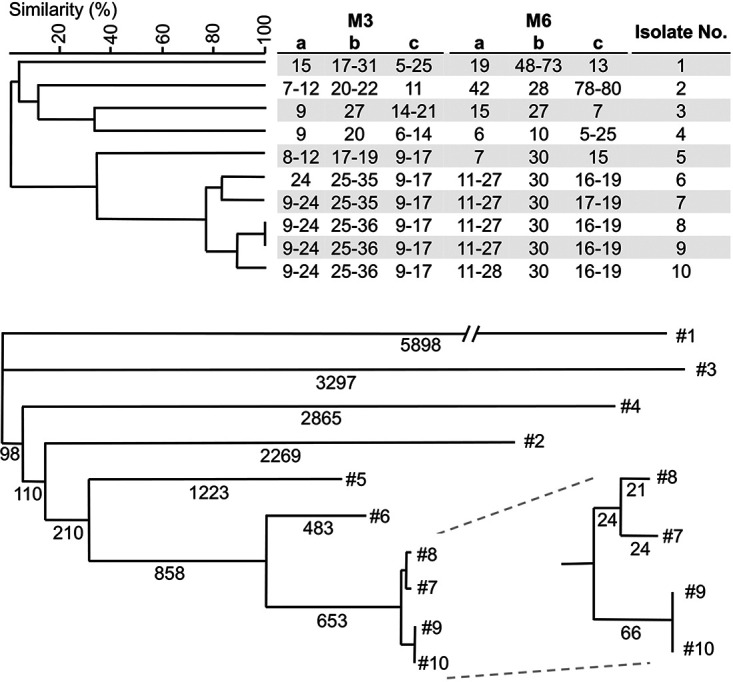
Comparison of genetic relatedness of 10 C. tropicalis isolates by STR typing (top) and WGS SNP typing (bottom). The UPGMA dendrogram was generated with BioNumerics. The numbers below the branches of the phylogenetic tree indicate the number of SNPs. The tree was generated with MEGA11 using the neighbor-joining tree method. Isolates were renamed to provide a clear overview, #1: 10-11-10-60, #2: 10-03-01-01, #3: 10-11-12-15, #4: 10-03-15-77, #5: 10-04-07-38, #6: 10-03-02-40, #7: 10-03-01-59, #8: 10-03-02-32, #9: 10-03-01-42, #10: 10-03-01-17.

To determine whether the genotypical relatedness found with STR typing correlated with the number of SNPs between strains, WGS of 10 isolates was performed. Reads from each strain were mapped to the IBUN-090-03567 reference genome, and homozygous SNPs between isolates were determined (Fig. 2). Isolates 1 to 6 differed by at least 2,500 SNPs, 4 or more STR markers, and originated form varying countries. Isolates that differed in at most 3 markers, exhibited <1203 SNPs between each other. Isolates 7 to 10 from Qatar, differed 0 to 114 SNPs, and were separated by at most 3 alleles. Isolates 8 and 9, which showed the same genotype according to STR, differed in 111 SNPs, while isolates 9 and 10 were identical according to SNP analysis, but were differentiated by STR analysis in 1 STR marker in 1 copy number. Copy numbers of repeats with a total length of <140 bp were all confirmed *in-silico* via comparison with WGS data (Table S3).

## DISCUSSION

This study describes the development of a novel C. tropicalis STR genotyping assay. The STR typing scheme consists of 2 multiplex PCRs, which together amplify 6 STR markers with a repeat size of 3 or 6 nucleotides, M3 and M6, respectively. STR typing of 117 C. tropicalis isolates identified 104 different genotypes. All markers were stable and specific for C. tropicalis, as copy numbers were not altered after subcloning 2 isolates for 100 generations, and no PCR products were formed for 15 other yeast species. WGS SNP analysis of 10 isolates was in agreement with STR typing.

### Application of C. tropicalis STR genotyping.

Application of C. tropicalis STR typing revealed the genetic relationship among strains isolated from patients in different countries. In total, 117 isolates were typed, of which 10 clusters containing 2 to 4 isolates were found (Fig. 1). Three clusters originated from Qatar, 1 from Kuwait, 1 from Greece, and 5 clusters from a single hospital in the Netherlands, for which epidemiological information was available. Each Dutch cluster contained multiple isolates from single patients with 2 closely related clusters containing isolates from the same patient. Isolates from these 2 clusters were all taken on the same day, and only differed 1 copy number in 1 marker. Another cluster contained 4 isolates with 17 months in between the first and last isolate collection, indicating a prolonged colonization by the same C. tropicalis strain. The remaining 2 clusters, constituting 2 and 3 isolates, were sampled over 3 and 2 days, respectively. Thus, isolates from the same patient were genetically related, while clusters from different patients were separated by STR, demonstrating that there was no C. tropicalis transmission within this hospital. To differentiate strains by STR typing, the absolute number of differing STR alleles was used, without taking the difference in copy numbers per marker into account. As such, the presence of a heterozygous or homozygous STR marker had an identical impact on relatedness as a STR copy number variation of 1. A challenge for diploid organisms is the potential perception of heterozygous alleles, as homozygous in the situation that SNPs in the STR primer binding region prevents amplification of 1 or 2 alleles, as was shown previously (26). Out of 117 isolates, 2 pairs of isolates exhibited identical STR profiles, except for a difference in zygosity in 1 marker. Another isolate pair differed in 2 markers in zygosity while demonstrating the same copy numbers for all other markers. WGS was performed on 1 isolate pair, which differed in 1 marker by zygosity, and in one other marker by 1 copy number. Visual inspection with JBrowse on WGS data confirmed the STR results, demonstrating that the potential false perception of heterozygous alleles as homozygous was not common in this study.

### Comparing genotyping methods.

In order to determine the resolution of the STR genotyping, 10 isolates were also investigated by WGS SNP analysis, and results were compared (Fig. 2). Five isolates from Qatar were highly related, according to STR typing, with small copy number differences in 3 markers at most, and were separated by <1170 SNPs. The 2 isolates with the same STR genotype differed by 111 SNPs. The other 5 isolates differed in 4 or more markers with much larger copy number differences, and originated from varying countries. Remarkably, 2 isolates from Qatar exhibited a different STR genotype, while being identical, according to SNP analysis. This could be explained by the potential high mutation rate of repeat regions, which are not included with SNP analysis, since they are indels compared to nonrepeating sequences (31). Based on this group of 10 isolates, we found that isolates that differed in 4 or more STR markers are also separated by thousands of SNPs, while isolates with an identical STR profile might differ up to 111 SNPs. This resolution should be considered preliminary, as WGS SNP analysis on more isolates with identical STR genotypes is necessary to determine the resolution of this STR assay more precisely. Furthermore, it is important to note that outcomes of different SNP calling pipelines can highly vary, due to different filtering steps.

When comparing different genotyping assays, the turn-around-time, costs, implementation potential, and discriminatory power are parameters that must be taken into account. Multiple MLST typing schemes have been developed for C. tropicalis (19, 21, 23). MLST approaches proved to be reproducible across varying laboratories, and can provide delineation of population structures, while having a low-turn-around-time. The application of MLST genotyping assays in outbreak settings remain questionable, due to the low mutation frequency of genes amplified in MLST schemes (23, 32–34). STR typing methods proved to be highly reproducible, and quickly executable with a high discriminatory power (25, 26, 35). Recently, 2 C. tropicalis STR typing schemes were developed by Wu et al. and Fan et al. (29, 30). The assay of Wu et al. was applied to 58 independent clinical C. tropicalis isolates, identifying 38 genotypes with 6 microsatellite markers (29). The assay of Fan et al. was used to type 82 different isolates from 32 patients, which identified 29 genotypes by amplifying 8 markers (30). Outcomes of these STR typing schemes have not been compared to WGS SNP analysis, which makes it difficult to determine the resolution, and its usefulness in outbreak settings. All microsatellite markers in both assays were amplified in monoplex PCRs, which is more expensive and time-consuming compared to amplifying markers in multiplex. The assay of Wu et al. reported no amplification for 7 out of 65 isolates for at least 1 marker, and its results were comparable with MLST genotyping to each other, showing a higher resolution than PCR-fingerprinting (29). The typing scheme of Fan et al. was compared to pulsed-field gel electrophoresis (PFGE). These methods yielded concordant results, although PFGE has a considerably longer turn-around-time, and poor comparability between laboratories (30). On average, the discriminatory power (DP) value for the typing of Wu et al. was 0.85, while the average DP value reported by Fan et al. was 0.89 (29, 30). Our STR loci demonstrated an average DP value of 0.91. Although our STR assay demonstrated the highest DP value, differences in discriminatory power between assays can be explained by the differences in the collection under investigation. Therefore, it does not necessarily mean that our resolution is best. The 6 STR markers in our assay were amplified in 2 multiplex PCRs, which leads to reduced costs and less hands-on time, while our STR results were also validated by comparison with WGS SNP analysis. Taken together, while our STR typing scheme has several advantages compared to other STR assays, comparison of identical isolates should determine which typing assay has the highest discriminatory power.

Altogether, we developed a specific, reproducible, and reliable STR typing method for C. tropicalis consisting of 2 multiplex PCRs, in which 6 markers are amplified. Typing of 117 C. tropicalis isolates identified 104 different genotypes. Genetic relatedness, according to STR of 10 isolates, correlated well with WGS SNP analysis while the STR typing is faster, less time-consuming, and less expensive than WGS.

## MATERIALS AND METHODS

### Isolates.

A total of 117 clinical C. tropicalis isolates were used in the STR typing scheme (Table S1). Prior to STR typing, the phylogenetic relationship between these isolates was unknown. Collection site of isolates were unknown, except for those from the Canisius Wilhelmina hospital in Nijmegen, The Netherlands. In addition, 15 other *Candida*, Cryptococcus, and *Saccharomyces* species were included to test the specificity of the STR assay (Table S2). Isolates were stored at –80°C, according to standard procedures. All isolates were identified by matrix-assisted laser desorption ionization-time of flight (MALDI-TOF) mass spectrometry, as previously described (36).

### Culture and DNA extraction.

All isolates were taken from storage at –80°C, and grown on Sabouraud agar plates (Oxoid) at 30°C. For STR genotyping and WGS, a single colony was resuspended in 400 μL MagNA Pure bacteria lysis buffer and MagNA Lyser green beads. These were mechanically lysed for 30 s at 6,500 rpm using the MagNA Lyser system (all Roche Diagnostics GmbH). DNA was extracted and purified with the MagNA Pure 96 instrument, the MagNA Pure DNA, Viral NA Small Volume Kit (Roche Diagnostics), following the manufacturer’s instruction. For WGS, all samples were subsequently treated with RNase at a final concentration of 5 μg/μL for 1 h at room temperature, after which DNA was extracted and purified with the MagNA Pure 96 instrument as described above. Purified DNA was measured with a Qubit 3.0 Fluorometer (Thermo Fisher Scientific), using the double-stranded DNA (dsDNA) high sensitivity option.

### Identification of STR loci.

The C. tropicalis reference genome IBUN-090-03567 (GCA_019239295.1) was downloaded from the NCBI database, and uploaded to the Tandem Repeats Finder (https://tandem.bu.edu/trf/trf.html) using the advanced search option (alignment parameter, 2,7,7; minimum alignment score to report repeat, 90; maximum period size, 10; maximum tandem repeat array size, 2) (37). The resulting STRs were screened, though repeats containing insertions or deletions, exhibiting a < 90% perfect match to the repeat sequence, or having a copy number of <6, were excluded. From the remaining STRs, 3 repeats with a period size of 3 nucleotides, and 3 repeats with a period size of 6 nucleotides were selected based on a high copy number (>20 and >10 for the trinucleotide and hexanucleotide, respectively) (Table S3).

### WGS.

Genomic libraries were prepared and sequenced with the Illumina NovaSeq 6000 platform (Illumina) with 2- by 150-bp paired-end-read mode at Eurofins Genomics (Ebersberg, Germany). Read data were uploaded to the Galaxy tool, and FastQC was used to assess the quality of the read data, and no trimming was performed (38). Read data were aligned against the C. tropicalis genome IBUN-090-03567 (GCA_019239295.1) using BWA-MEM (39). Read duplicates were removed using RmDup, local realignment was performed with BamLeftAlign, and unpaired reads were removed with BAM filter. Mapped reads with a MAPQ score <60 were removed. WGS alignments of the 6 selected STRs and flanking sequences were visually inspected using JBrowse v1.16.11 to identify variants in primer binding sites (40).

### Primer design, PCR, and genotyping.

Primers were designed with Primer3Plus (https://www.bioinformatics.nl/cgi-bin/primer3plus/primer3plus.cgi), using default settings, except for primer size (minimum [Min], 19; optimum [Opt], 21; maximum [Max], 24) primer melting temperature (T_m_) (Min, 57.0; Opt, 59.0; Max, 62.0), Max-Poly-X (3), and GC clamp (1) (41). Variants in STR flanking sequences were marked as excluded regions. Primers that formed no self- or cross-dimers with 5 or more nucleotides from the last 7 nucleotides of the 3′ end of a primer, according to the multiple-primer analyzer from Thermo Fisher Scientific, were ordered via Eurogentec. Multiplex PCR was performed on a thermocycler (Biometra) using 1x FastStart *Taq* polymerase buffer without MgCl_2_, deoxynucleoside triphosphates (dNTPs) (0.2 mM), MgCl_2_ (3 mM), forward (fwd) and reverse (rev) primers (1 to 5 μM), 1 U FastStart *Taq* polymerase (Roche Diagnostics), and isolated DNA. A thermal protocol of 10 min of denaturation at 95°C, followed by 30 cycles consisting of 30 s denaturation at 95°C, 30 s of annealing at 60°C, and 1 min of extension at 72°C with a final incubation step for 10 min at 72°C was used for PCR amplification. PCR products were diluted 1:1,000 in water, and 10 μL together with 0.12 μL of the Orange 600 DNA size standard (NimaGen) were incubated for 1 min at 95°C, and analyzed on a 3500 XL genetic analyzer (Applied Biosystems).

### Data analysis and discriminatory power.

Copy numbers of all 6 STR markers were determined using GeneMapper 5 software (Applied Biosystems). For all markers, stutter peaks lower than 50% of the intensity of the highest peak for an allele, minus-A peaks and blead-through peaks, were discarded. Subsequently, the size for the STR allele was rounded. Copy numbers for repeats were converted to a binary matrix: 1 if an isolate contained the allele, and 0 if it did not. Relatedness between isolates was analyzed using BioNumerics software version 7.6.1 (Applied Maths) via the unweighted pair group method with arithmetic means (UPGMA), using the multistate categorical similarity coefficient. The discriminatory power of the STR analysis was determined using the Simpson index of diversity (D), as described previously (35). STR copy numbers of sequenced isolates were *in-silico* validated by visualization of repeat regions in JBrowse v.1.16.11.

### WGS SNP calling.

Homozygous SNPs in all isolates were detected with Freebayes using the default settings except for allelic scope options (ignore indels, multiple nucleotide polymorphisms [MNPs], and complex events) (42). Resulting SNPs in the VCF file with a read depth (DP) of <25, a quality (QUAL) of <100, an allele frequency (OA) of <0.15 x DP, and an AO > 0.90 x DP were removed. Phylogenetic analysis was performed with VCF2PopTree, using the genetic drift algorithm, and a MEGA distance-based matrix was developed (43). The matrix was uploaded to MEGA11, and a phylogenetic tree was generated using the Neighbor-joining Tree method (44). The SNP calling pipeline was previously validated with a Candida auris benchmark data set (45, 46).

### Data availability.

All raw read data generated in this study has been deposited to the National Center for Biotechnology Information’s Sequence Read Archive (BioProject ID: PRJNA833395). Read sequences of all strains are deposited under the following accession numbers: SRR18971072, SRR18971071, SRR18971070, SRR18971069, SRR18971068, SRR18971067, SRR18971066, SRR18971065, SRR18971064 and SRR18971063.
