# Supplementary material for: Development and Application of a Short Tandem Repeat Multiplex Typing Assay for Candida tropicalis
Source: Microbiol Spectr. 2023 Jan 30;11(2):e04618-22. doi: 10.1128/spectrum.04618-22 (PMC10100945; doi:10.1128/spectrum.04618-22)
Supplement: Supplemental file 1 — Fig. S1 and Tables S1 to S3. Download spectrum.04618-22-s0001.pdf, PDF file, 0.8 MB [file spectrum.04618-22-s0001.pdf]

Figure S1: Cluster analysis of the 117 *C. tropicalis* isolates from varying countries. Branch lengths indicate relatedness according to microsatellite alleles. The UPGMA dendrogram was generated with BioNumerics.

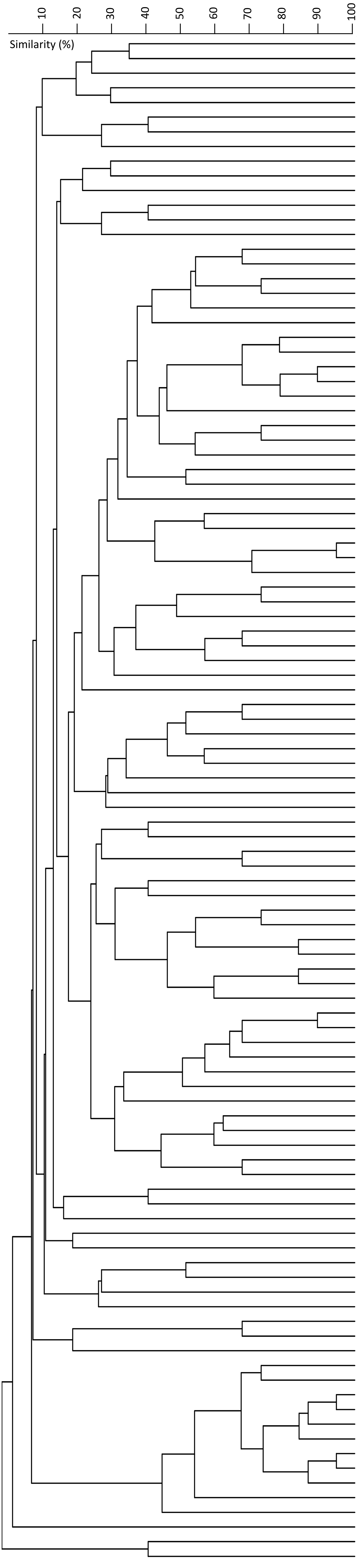

M3			M6			Genotype	No. of isolates	Country
a	b	c	a	b	c			
8/12	17/19	9/17	7	30	15	1	1	Unknown
8/12	20/39	9/16	23	29	16/19	2	1	Qatar
8/13	31/35	5/16	7	30	10/52	3	1	Unknown
8/24	19	17	6/10	25	13/15	4	1	India
7/8	19/35	9	10/34	28	16/52	5	1	Qatar
8	17	8/10	4/7	73	5/18	6	1	Unknown
8	17	20/23	7/9	22/28	10/18	7	1	Qatar
8/14	24/26	10/13	7/8	24/28	18/63	8	1	Unknown
7/21	24/28	24	3/10	29/39	12	9	1	India
15/21	17	9	3/7	29	14/50	10	1	Netherlands
7/14	8/20	9/15	7/10	3	11	11	1	Qatar
7/9	17/25	6	16	25	13/34	12	1	Qatar
7/15	17/25	6	3/7	73	7/20	13	1	Qatar
15	17/31	5/25	19	48/73	13	14	1	Netherlands
9	28	10	3/11	26	8	15	1	India
9	17	10	3/9	27	8	16	1	India
9	27	10	6/8	27/30	8	17	1	India
9	27	10	6/43	27	79	18	1	India
13	27	10	6/9	26	8/15	19	1	India
8	25	10	6/8	28/45	15	20	1	India
9	27	14/16	3/10	26	7	21	2	Qatar
9	27	14	3	24	7	22	2	Netherlands
9	27	14/21	15	26	7/15	23	3	Netherlands
9	27	14/21	16	26	7/15	24	1	Netherlands
9	27	14/21	15	27	7	25	1	Netherlands
9/15	27	16	18/20	29	7/14	26	1	Qatar
9	27	6	3	39	9	27	1	Qatar
9	27	6	12	3/31	9	28	1	Qatar
7	27	6	8	22	19	29	1	Qatar
9	31	6	31/36	29/32	10	30	1	Qatar
9	28	6	15	28	8/10	31	1	Unknown
9/15	22/27	9	8	24	22/77	32	1	Netherlands
9	25/28	6/10	3	18	14/45	33	1	Netherlands
9	25/28	6/10	12	10/32	16	34	1	Netherlands
9	18/28	10	12/14	28	15/17	35	2	Netherlands
9	18/28	10/18	12/14	28	15/17	36	2	Netherlands
9	18/28	10/18	12	10/18	17	37	1	Greece
9	17/20	9	10	59	24	38	1	Unknown
9	17/20	9	3/16	59	5	39	4	Netherlands
9/22	20	9	7	28/35	5/23	40	1	Qatar
8/10	20	6/14	6	28	5	41	1	Netherlands
9	20	6/14	6	10	5/25	42	1	Greece
8	20	6/14	8	45/59	5/15	43	1	Netherlands
3	17	14	6/8	10	7/24	44	1	Netherlands
8/9	26/28	6/12	3/6	10	14/63	45	1	Qatar
9	30	13	9	24/27	13	46	1	India
9/13	28/31	13	7	24/27	13	47	1	India
9/13	28/31	9/14	9	27	68	48	1	Qatar
9/14	28/31	9/13	7	27	10/63	49	1	Qatar
9/13	31/35	9/13	7	30	10/19	50	1	Netherlands
9/15	28/31	13/44	4/13	27/29	13/30	51	1	India
14	29/31	9/13	12/16	25/27	80	52	1	Qatar
9/14	28/33	9/13	3	28	9/41	53	1	Qatar
7	15/20	10	7	10/18	30	54	1	Qatar
7/8	15/28	6/10	7	25/31	9/39	55	1	Qatar
7	29/30	10	8/45	39	42/43	56	1	India
7/12	28	10	8/45	39	30	57	1	India
7/14	28/45	12	6	27	37	58	1	India
6/11	28	10	7/8	27	13/15	59	1	India
7/21	17/28	12	7/8	39	19	60	1	India
7/21	17/28	12/24	7/8	28/29	17/19	61	1	India
7/21	25/28	12/24	7/8	30	20/37	62	1	India
7/21	25/28	12	7/8	25	20/36	63	1	India
7	28	12	7	25/26	15	64	1	India
7	28	12	7/8	25/26	8/15	65	1	India
7/12	20/29	12	7/36	25	15	66	1	India
7/9	25/28	9	8	10	10/19	67	1	Qatar
7/9	25/29	9	8	10	10/19	68	1	Qatar
7/9	25/28	9	8/35	18	9/19	69	1	Kuwait
7/9	25/28	9	4/36	30	10/19	70	1	Qatar
9	17/18	9	8	10/32	10	71	1	Qatar
7	25/28	9	38/40	10/32	10/20	72	1	Qatar
7/12	15/28	6/9	8	10/28	5/52	73	1	Qatar
7/12	20/28	11	42	28	78/80	74	1	Qatar
7/12	20/28	11	45	10	88	75	2	Greece
7	20/28	11	7	26	10	76	1	Netherlands
13/14	28	11/19	8	10	10/59	77	1	Kuwait
13	28	11/19	8	18	10/31	78	1	Netherlands
8/15	20/27	11	15/33	26	11/13	79	1	India
8	20/27	11	7/8	28/48	19	80	1	India
9	20/28	11/25	8/46	30/31	17/42	81	1	India
9/20	29	10/13	38/40	30	21/28	82	1	Netherlands
8/20	22/25	10/36	18	10	11	83	1	Qatar
7/12	28	10	14	27/31	22	84	1	India
9/24	17	13	8/14	27/31	22	85	1	India
24/28	7/21	12/26	3/29	27/31	11/22	86	1	India
7/9	21/29	13/24	3/9	27/29	11/22	87	1	India
7/12	20/29	12/21	6	27/30	8	88	1	India
7/12	20/29	12/21	3/9	23	8	89	1	India
10/12	20/29	5/9	14/23	33	29/49	90	1	Qatar
9/24	25/35	9/17	11/27	30	17/19	91	1	Qatar
9/24	25/36	9/17	11	27	17/19	92	1	Qatar
9/24	25/36	9/17	11/27	30	16/19	93	2	Qatar
24	25/35	9/17	11/27	30	16/19	94	1	Qatar
9/24	25/36	9/17	11/28	30	16/19	95	2	Qatar
9/24	25/36	9/17	11/27	10	16/19	96	1	Qatar
9/24	25/41	9/17	27	30	16/20	97	1	Qatar
9/24	25/41	9/17	11/27	30	16/20	98	1	Qatar
9/24	25/41	9/17	11/27	30	16/19	99	1	Qatar
9/24	25/39	9/17	12/29	10	16/19	100	1	Qatar
9/25	28/41	9	3/27	10	16/19	101	2	Kuwait
7/12	28/32	5/9	21/36	18/27	9/35	102	1	Qatar
12	25/34	10/24	4/13	25/26	13/30	103	1	India
12/16	25/34	10/24	6/43	27	65/71	104	1	India

Table S1: Overview of all *C. tropicalis* samples used for STR typing.

CWZ-ID	Alt. ID	Country	City	Genotype
10-04-07-38	YFJ002-127	Unknown		1
10-03-02-90	189	Qatar		2
10-07-05-85	5081350175	Unknown		3
42202		India	Chandigarh	4
10-03-01-94	094	Qatar		5
10-06-16-45	4012981937	Unknown		6
10-03-02-67	166	Qatar		7
10-06-04-28	1092268359	Unknown		8
42186		India	Changdigarh	9
10-06-16-80	4021989610	The Netherlands	Nijmegen	10
10-03-03-06	204	Qatar		11
10-03-01-97	097	Qatar		12
10-03-02-30	129	Qatar		13
10-11-10-60	2120432052	The Netherlands	Nijmegen	14
42193		India	Chandigarh	15
42199		India	Chandigarh	16
42195		India	Chandigarh	17
42200		India	Chandigarh	18
42213		India	Chandigarh	19
42212		India	Chandigarh	20
10-03-02-75	164	Qatar		21
10-03-03-17	215	Qatar		21
10-06-06-38	2022220919	The Netherlands	Nijmegen	22
10-06-06-39	2022521378	The Netherlands	Nijmegen	22
10-06-12-56	3043089736	The Netherlands	Nijmegen	23
10-06-12-71	3051393264	The Netherlands	Nijmegen	23
10-06-19-82	4082548606	The Netherlands	Nijmegen	23
10-06-13-37	3061913500	The Netherlands	Nijmegen	24
10-11-12-15	5012088590	The Netherlands	Nijmegen	25
10-03-03-03	201	Qatar		26
10-03-01-38	038	Qatar		27
10-03-02-92	191	Qatar		28
10-03-02-65	164	Qatar		29
10-03-01-46	046	Qatar		30
10-07-04-83	5060430872	Unknown		31
10-07-03-72	5040816007	The Netherlands	Nijmegen	32
10-12-14-29	9102851254	The Netherlands	Nijmegen	33
10-06-10-35	2111322974	The Netherlands	Nijmegen	34
10-06-09-07	2090792426	The Netherlands	Zwolle	35
10-06-09-10	2090792422	The Netherlands	Zwolle	35
10-06-09-08	2090792423	The Netherlands	Zwolle	36
10-06-09-09	2090792424	The Netherlands	Zwolle	36
10-12-12-06		Greece		37

CWZ-ID	Alt. ID	Country	City	Genotype
42205		India	Chandigarh	51
10-03-02-44	143	Qatar		52
10-03-03-28	226	Qatar		53
10-03-01-54	054	Qatar		54
10-03-02-61	160	Qatar		55
42183		India	Chandigarh	56
42208		India	Chandigarh	57
42197		India	Chandigarh	58
42206		India	Chandigarh	59
42182		India	Chandigarh	60
42184		India	Chandigarh	61
42188		India	Chandigarh	62
42190		India	Chandigarh	63
42192		India	Chandigarh	64
42194		India	Chandigarh	65
42198		India	Chandigarh	66
10-03-01-60	060	Qatar		67
10-03-01-77	077	Qatar		68
10-08-13-91	Kw1020/16	Kuwait		69
10-03-01-93	093	Qatar		70
10-03-01-40	040	Qatar		71
10-03-02-46	145	Qatar		72
10-03-02-20	119	Qatar		73
10-03-01-01	001	Qatar		74
10-05-12-97		Greece		75
10-05-14-30		Greece		75
10-07-02-12	5012088590	The Netherlands	Nijmegen	76
10-08-13-95	Kw107/17	Kuwait		77
10-06-08-10	2062462448	The Netherlands	Nijmegen	78
42185		India	Chandigarh	79
42211		India	Chandigarh	80
42189		India	Chandigarh	81
10-08-14-24	7052948274	The Netherlands	Nijmegen	82
10-03-01-65	065	Qatar		83
42203		India	Chandigarh	84
42210		India	Chandigarh	85
42209		India	Chandigarh	86
42207		India	Chandigarh	87
42196		India	Chandigarh	88
42204		India	Chandigarh	89
10-03-01-39	039	Qatar		90
10-03-01-59	059	Qatar		91
10-03-02-96	195	Qatar		92

10-06-08-42	2071872814	Unknown		38
10-06-18-31	4052823800	The Netherlands	Nijmegen	39
10-06-19-02	4071338087	The Netherlands	Nijmegen	39
10-07-02-01	5011486859	The Netherlands	Nijmegen	39
10-07-02-04	5011486854	The Netherlands	Nijmegen	39
10-03-01-61	061	Qatar		40
10-03-15-77		Greece		42
10-06-10-39	2111524144	The Netherlands	Nijmegen	43
10-06-13-85	3072724550	The Netherlands	Nijmegen	44
10-03-01-21	021	Qatar		45
42214		India	Chandigarh	46
42215		India	Chandigarh	47
10-03-02-72	171	Qatar		48
10-03-01-18	018	Qatar		49
10-07-07-02	5101468409	The Netherlands	Nijmegen	50
10-03-01-41	041	Qatar		93
10-03-02-32	131	Qatar		93
10-03-02-40	139	Qatar		94
10-03-01-02	002	Qatar		95
10-03-01-17	017	Qatar		95
10-03-01-45	045	Qatar		96
10-03-02-14	113	Qatar		98
10-03-01-37	037	Qatar		99
10-03-02-77	176	Qatar		100
10-08-13-92	Kw3005/16	Kuwait		101
10-08-13-94	Kw3029/16	Kuwait		101
10-03-01-80	080	Qatar		102
42191		India	Chandigarh	103
42201		India	Chandigarh	104

Table S2: Related yeast species and outcome of specificity testing.

**Species**	**M3**			**M6**		
	**a**	**b**	**c**	**a**	**b**	**c**
Candida albicans	-	-	-	-	-	-
Candida parapsilosis	-	-	-	-	-	-
Candida krusei	-	-	-	-	-	-
Candida dubliniensis	-	-	-	-	-	-
Candida guilliermondii	-	-	-	-	-	-
Candida lusitaniae	-	-	-	-	-	-
Candida auris	-	-	-	-	-	-
Candida haemulonii	-	-	-	-	-	-
Candida glabrata	-	-	-	-	-	-
Candida kefyr	-	-	-	-	-	-
Candida orthopsilosis	-	-	-	-	-	-
Candida pelliculosa	-	-	-	-	-	-
Cryptococcus neoformans	-	-	-	-	-	-
Saccharomyces cerevisiae	-	-	-	-	-	-

Table S3: *In-silico* validation of STR copy numbers of 11 sequenced isolates. Copy numbers demarcated in bold were visually validated with WGS data

Isolate ID	M3				M6		
	a	b	c		a	b	c
10-03-01-41	**9/24**	**25/37**	**9/17**		**11**/27	30	**16/19**
10-03-02-32	**9/24**	**25/37**	**9/17**		**11**/27	30	**16/19**
10-03-02-40	**24**	**25/37**	**9/17**		**11**/27	30	**16/19**
10-03-01-02	**9/24**	**25/37**	**9/17**		**11**/28	30	**16/19**
10-03-01-17	**9/24**	**25/37**	**9/17**		**11**/28	30	**16/19**
10-03-01-59	**9/24**	**25/36**	**9/17**		**11**/27	30	**17/19**
10-04-07-38	**8/12**	**17/19**	**9/16**		**7**	**7**/30	**15**/37
10-11-12-15	**9**	**27**	**14/21**		**17**	24/30	**21**/28
10-03-15-77	**9**	**20**	**6/14**		**6**	**6/10**	**5**/25
10-11-10-60	**35**/43	**12/33**	**19**		**19**	**3**	**13**
10-03-01-01	**7/12**	**20/28**	**11**		42	28	71/80
